# COVID/HIV Co-Infection: A Syndemic Perspective on What to Ask and How to Answer

**DOI:** 10.3389/fpubh.2021.623468

**Published:** 2021-03-10

**Authors:** Hailay Abrha Gesesew, Lillian Mwanri, Jacqueline H. Stephens, Kifle Woldemichael, Paul Ward

**Affiliations:** ^1^Flinders Health and Medical Research Institute, Flinders University, Adelaide, SA, Australia; ^2^Epidemiology, School of Health Sciences, Mekelle University, Mekelle, Ethiopia; ^3^Epidemiology, Institute of Health, Jimma University, Jimma, Ethiopia

**Keywords:** HIV, COVID-19, syndemic approach, framework, HIV care continuum

## Abstract

The present commentary explored the intersecting nature of the COVID-19 and HIV pandemics to identify a shared research agenda using a syndemic approach. The research agenda posits the following questions. Questions around HIV infection, transmission, and diagnosis include: (i) molecular, genetic, clinical, and environmental assessments of COVID-19 in people living with HIV, (ii) alternative options for facility-based HIV testing services such as self- and home-based HIV testing, and (iii) COVID-19 related sexual violence and mental health on HIV transmission and early diagnosis. These and related questions could be assessed using Biopsychosocial and socio-ecological models. Questions around HIV treatment include: (i) the effect of COVID-19 on HIV treatment services, (ii) alternative options for facility-based treatment provision such as community-based antiretroviral therapy groups, and (iii) equitable distribution of treatment and vaccines for COVID-19, if successful. Bickman's logic model and the social determinants of health framework could guide these issues. The impact of stigma, the role of leveraging lessons on sustained intra-behavioral change, the role of medical mistrust and conspiracy beliefs, and the role of digital health on integrated management of HIV care and spectrum of care of COVID-19 need assessment using several frameworks including Goffman's stigma framework, Luhmann's Trust theory, and Gidden's theory of structuration. In conclusion, the potential research agenda of this commentary encompasses a variety of research fields and disciplinary areas—clinicians, laboratory scientists, public health practitioners, health economists, and psychologists—, and suggests several theoretical frameworks to guide examination of complex issues comprehensively.

## Introduction

The human immunodeficiency virus (HIV) (1) and novel coronavirus disease 2019 (COVID-19) (2) pandemics have some similarities. Both diseases are caused by a virus and currently do not have a vaccine or a cure. HIV is primarily transmitted through unsafe sex, blood contact, and mother-to-child transmission, whereas COVID-19 is transmitted through droplets and direct contact. Both conditions may initially present with influenza-like symptoms, such as fever, cough, and difficulty in breathing, although the severity and clinical stage varies (3).

Clinically, patients with COVID-19 have been reported to have one of the following five outcomes (3): asymptomatic (1.2%), mild to moderate symptoms (80.9%), severe (13.8%) and critical conditions (4.7%), and death (2.3%). Some population groups, including people who are older (and male), consume alcohol, have one or more comorbid condition, and live in densely populated settings such as in refugee camps, are at a higher risk of severe COVID-19 related infection or death. Similarly, HIV transmission is high in refugee camps and among people who consume excessive alcohol (4, 5), and AIDS-related mortality is high among older populations and people with one or more comorbidities. Safe sex and use of sterile injection equipment are common prevention methods of HIV transmission. Early case detection, isolation of confirmed cases, quarantine, contact tracing, social distancing, hand washing, and use of alcohol-based sanitizer and personal protective equipment (PPE) are techniques implemented to reduce the risk of COVID-19 transmission.

Given the elements of both COVID-19 and HIV discussed above, a syndemic assessment of the spectrum of both infections may benefit HIV, COVID-19, or COVID-19/HIV co-infected patients. A brief literature review on “HIV” and “COVID-19” has shown some commonalities and interactions between COVID-19 and HIV. These include an increased burden of COVID-19 in people living with HIV (6, 7), increased burden of COVID-19 and HIV in migrant workers (8), increased burden of COVID-19 and HIV in sex workers (9), and increased burden of COVID-19 and HIV in men who have sex with men (10). Mhango et al. indicated COVID-19 lockdowns have impacted facility-based HIV testing and suggest the need to scale up home-based HIV testing in sub-Saharan Africa (11). Additionally, better clinical outcomes of COVID-19 in people living with HIV have been reported (12–15), provoking a debate whether lessons learned from the HIV response can inform effective response to mitigate COVID-19 (16–20). At this stage, we are cognisant the evidence about the interactions, impacts, and synergy between the spectrum of COVID-19 and continuum of HIV care is still building.

The aim of this article is therefore to highlight the potential syndemic perspectives of COVID-19 and HIV pandemics systematically. In particular, it will focus on posing research questions around biomedical, behavioral, psychosocial, and structural issues of COVID/HIV co-infection and potential theoretical frameworks to investigate these questions. This commentary also explores the implications for research in sub-Saharan Africa. Ward has published a research agenda of COVID-19 for sociologists (21) and Holmes et al. on COVID-19 and mental health sciences (22). Our commentary will pose broader research questions, through a syndemic perspective, for clinicians, laboratory technologists, public health practitioners, health economists, psychologists, and sociologists which were not covered in the aforementioned research agendas. Such syndemic framework presents a meaningful and robust paradigm to raise questions on the potential benefits and impact of co-designing COVID/HIV health programming services which can tackle the two pandemics concurrently. While the aim of this article is to ask a myriad of questions and suggest several frameworks, synthesizing lessons learned from the successes and failures of the HIV pandemic journey is vital. We are aware of evidence demonstrating successful lessons of HIV care to control other non-communicable chronic diseases (23, 24), and we argue there could be a potential research agenda to curb both HIV and COVID-19 based on the cascades of HIV care.

## Discussion

### Questions Around HIV Infection, Transmission, and Diagnosis

Given COVID-19 facts are new and emerging (only 8 months at the time of writing), the biomedicine of COVID-19, especially about its interaction with HIV, is a new field. The biomedical component of Engel's biopsychosocial model (25) can frame the clinical, analytical, and radiological presentation of COVID-19 in HIV-infected individuals. Specifically, the pathological interaction between the severe acute respiratory syndrome coronavirus-2 (SARS-COV-2) and HIV needs further exploration given its implication for therapeutic and vaccine development. Until this stage of the COVID-19 pandemic, there have been differences in outcomes of COVID-19 patients between people living in Africa (Africans) and people living elsewhere, such as in Europe. For example, the mortality rate in USA, Spain and Italy—the most COVID-19-affected countries in April 2020—were 4.6, 10.5, and 13.1%, respectively, whereas the mortality rates in the most affected African countries in April 2020 were 1.8% in South Africa, 7.2% in Egypt, and 5.3% in Morocco (26). Some explanations about these differences have been related to the differences with high and low exposure to microorganisms and parasites among Africans and Europeans, respectively (27), and the affluence-related travel in Western countries. Debate on these issues has never reached consensus, but the clear differences in the activation, pro-inflammatory, and memory profiles of the immune cells among Africans vs. Europeans require detailed investigation particularly on molecular, genetic, and environmental assessment. The assessment of the role of “trained immunity” and virtual memory T-cells in defending SARS-COV-2, and what this looks like among people living with HIV is also vital. Furthermore, the role of non-biological factors, such as less movement via air traffic and political motivations of countries to “under report” in sub-Saharan Africa, could be an additional research agenda. This will solve why Africa is the least affected continent with COVID-19 so far, given the continent has significant inequities, such as being densely populated, many people living in slum areas, a fragile health care system, a high prevalence of other infectious diseases, low literacy, a highly communal population, a significant proportion of the population living in poverty, and limited access to water (27).

Across the globe, health care services have been interrupted by COVID-19 related measures, such as lockdown or curfew. For example, the interruption of facility-based HIV testing poses a range of questions, including: (i) how does the COVID-19 pandemic affect facility-based HIV testing?, (ii) would the COVID-19 pandemic necessitate self-HIV testing and/or home-based HIV testing as an alternative option?, (iii) in the context of COVID-19, what would the economic evaluation of self-HIV testing and homebased HIV testing be compared to the facility-based HIV testing?, and (iv) how can COVID-19 related contact tracing be integrated within the self- or home-based HIV testing? These questions could be assessed at individual, community, health institution, and policy levels using the socio-ecological model.

COVID-19 could also increase the risk of HIV transmission as a result of COVID-19 related lockdowns. Already, evidence shows an increase in COVID-19 related sexual violence (28, 29) and poor mental health (30, 31), a point which could be probed using Engel's psychosocial model (25). Furthermore, the consumption of alcohol has been reported to have increased during the lockdown period (32), which in turn could exacerbate violence and subsequently HIV transmission. Hence, the unintentional impact of COVID-19 public health measures on HIV transmission and early diagnosis needs to be explored, including quantifying the number of new people living with HIV as a result of lockdown. While HIV can be transmitted through breastmilk, there is no evidence for transmission of COVID-19. However, as the knowledge about COVID-19 is still emerging, this would be an area for the research agenda.

### Questions Around HIV Treatment, Linkage, and Retention

COVID-19 disrupts HIV treatment services, but questions on how it impacts the collection and follow up of antiretroviral therapy (ART) drugs, prevention of mother-to-child transmission (PMTCT) services, monthly ART meetings, and ART training services is yet to be investigated. In many settings, the distribution of HIV promotion, prevention, testing, and treatment logistics have been disrupted because resources to support HIV have been shifted to mitigate the COVID-19 pandemic (33, 34). The impact of these, and related issues, as well as how to address these, need further research. Lockdowns and other public health measures have interrupted facility-based ART care services and further examination is needed if community-based ART groups, “pick and run strategy,” and appointment spacing model could be alternative options. Additionally, the economic evaluation of community-based vs. facility-based ART provision, and cost of HIV treatment among people living with HIV or COVID/HIV co-infection need additional investigation. The program theory or logic model conceptualized by Bickman has a number of elements (35) which could guide the exploration of these research questions.

The ongoing ART and combination therapy trials may be the focus of treatment attention. Executing clinical trials and combination therapies could resolve the existing ambiguity between the severity of COVID-19 on immunosuppressed patients (e.g., people living with HIV) vs. the potential effects of HIV antivirals in suppressing SARS-COV-2 replication. Furthermore, the COVID-19 pandemic could also be the opportunity for a research agenda to explore untapped areas, such as the four decades of unsuccessful searching for a HIV cure. Should COVID-19 treatment or vaccine be successful, strategies would also need to be explored to address their cost-effectiveness and equitable distribution. The framework on social determinants of health guides the elements of equitable distribution (36, 37). This will be an essential research agenda, given the lessons learnt from the arrival of anti-tuberculosis therapies in Africa after 35 years (38) and HIV treatment after 10 years (39, 40) of use in the developed west.

### Questions Around HIV Treatment Outcomes and Impacts

HIV treatment outcomes among people living with HIV co-infected with chronic diseases, such as tuberculosis, diabetes mellitus, hypertension, and other cardiac diseases, have been described elsewhere (41–49). The negative outcomes across the whole HIV care continuum (50) (i.e., late HIV diagnosis, late presentation to ART care, adherence to and lost-to-follow-up from ART, and clinical, immunological and virological failures) among COVID/HIV co-infected patients and people living with HIV is yet to be comparatively investigated. Such investigation would contribute to the success or failure of the 2030 UNAIDS 95-95-95 treatment targets (51), where 95% of people living with HIV would know their HIV status, 95% of people who know their status would receive treatment, and 95% of people on HIV treatment would have a suppressed viral load. The social determinants of health, such as poverty, gender, low literacy, racial, or sexual minority, immigrants, commercial sex workers, homelessness, and mental health would also need descriptive, inferential, and explorative investigations to find out how they relate with COVID/HIV co-infected patients. The social determinants of health framework by the World Health Organization (WHO) (36) and Baum et al. (37) would provide a guiding framework to comprehensively address these questions.

COVID-19 related stigma is also on the rise (52–54). The health disparity resulting from double, triple, or sometimes quadruple burden and stigma should comprehensively be studied. For example, consistent with other inequities, it would be interesting to explore the COVID/HIV health burden in vulnerable populations, such as poor black migrant women in developed countries. Stigma is a cross-cutting barrier and needs new strategies, including virtual methodologies and other digital health interventions, to halt its multidimensional impact. Goffman's stigma framework (55) and other revised versions (56, 57) could be a starting point of assessment. In relation to this, the quality-adjusted life years (QALYs) and disability-adjusted life years (DALYs) (58) of COVID/HIV co-infected patients need economic evaluation. The WHO quality of life framework (59) would help the exploration of the different components of quality of life, as well as to estimate the QALYs and DALYs.

### Questions Around HIV Care Promotion and Prevention Services

Given HIV does not currently have either a vaccine or curative therapy, sustained behavioral change is the main method used to substantially influence positive outcomes of HIV care and treatment. Lessons on the assessment of how sustained behavioral change at the intrapersonal level could be achieved [e.g., by health belief model (60)] would need further investigation among HIV/COVID-infected patients. Equally important, the contribution of medical mistrust and conspiracy beliefs, as described by Luhmann (61) and Giddens (62), and more recently by Ward (63–65) can undermine data-driven public health interventions. Beyond the intrapersonal issues, the integrated role of peer educators or community health workers for the prevention and promotion of HIV and COVID-19 could be vital. Given 80% of people in Africa visit traditional healers, the collaboration of traditional and modern HIV care providers could also be profoundly essential to managing both illnesses. The structuration model of collaboration (66) which has governance, formalization, internalization, and shared goals and vision dimensions, along with Gidden's theory of structuration (67), could be used to guide the elements of collaboration between the modern and traditional health practitioners.

Vaccine and therapeutic studies for the COVID-19 pandemic have been a research agenda since its declaration, but as yet have not led to successful outcomes. However, as of 30 October 2020, there were more than 2,434 therapeutic and 271 vaccine trials for COVID-19 registered in ClinicalTrials.gov. The fight to realize the rollout of vaccines against COVID-19 and the level of efficacy should be observed continually with the hope of achieving success in the near future. If successful, the cost of the vaccine per dose and the equitable distribution will be an important issue for scrutiny, in particular by drawing on lessons from the arrival of the BCG vaccine in Africa, 50 years after its original use in Europe and USA (68). It is imperative a time-lag does not occur after the implementation of a COVID-19 vaccine in Europe and the USA. Additionally, an agenda should be set to explore the potential implication of the UK's 340 million doses pre-order of four different vaccine types for COVID-19 (69), USA's 100 million COVID-19 vaccine doses pre-order (and agreement to procure an additional 500 million doses) from two vaccine companies, and charging extra health insurance for Americans even if it is said that “*the COVID-19 vaccine will be made available to Americans at no cost*” (70).

### Cross-Cutting Questions Around HIV and COVID-19 Care Services

The role of digital health in the integrated management of the HIV care continuum and spectrum of care of COVID-19 needs special attention, given the current and ongoing use of technology in health-related services. The application of technology on data collection for COVID-19 related research, potential biases and how to address them; the expansion and innovation of mobile applications for contact tracing (71) in Singapore, South Korea, Australia, and other countries; and the use of social media for research findings dissemination are some additional avenues for research.

The COVID-19 pandemic has so far caused unprecedented health, economic, and social impacts globally, with Europe and USA being the most affected regions, while other locations have responded well, such as South Korea who even conducted an election successfully during the first wave of the pandemic (72). Even within countries there is strong evidence of differing pandemic responses and the impact this has on risk mitigation. For example, in Australia the community-driven response by Aboriginal Community Controlled Health Organizations was initiated in a timely, clear and culturally appropriate way resulting in a highly successful response for Aboriginal and Torres Strait Islander Peoples (73, 74). This community-led leadership, which occurred separate to government-driven leadership and messaging, has resulted in very low levels of COVID-19 among this at-risk population (75). Such disparities in COVID-19 pandemic outcomes between regions and communities pose questions about the impact of leadership on COVID-19 and crisis management, and the performance of countries to mitigate the pandemic and its complications in general. The performance of low- and middle-income countries who performed well in mitigating the COVID-19 pandemic i.e., they are “punching above weight,” and developed countries who performed less well i.e., “punching below weight” needs further investigation. Baum et al. (37) proposed a framework on how to compare punching above and below weight countries (37).

Table 1 presents the summary of research agenda and respective theoretical frameworks.

**Table 1:** Summary of research questions and frameworks to address COVID/HIV co-infection.

**Theme**	**Research agenda**	**Theoretical framework or model**
HIV infection, transmission, and diagnosis	What are the clinical, analytical, and radiological presentations of COVID-19 in HIV-infected individuals?	Biological component of Biopsychosocial model (25)
	What are the molecular, genetic and environmental assessments of African vs. European COVID-19 patients look like?	
	How does COVID-19 pandemic affect the facility-based HIV testing? Any alternative options such as self-HIV testing and homebased HIV testing? And are they cost-effective?	Socio-ecological model
	How can COVID-19 related contact tracing be integrated within the self- or home-based HIV testing?	
	What are the roles of COVID-19 related sexual violence and mental health in HIV transmission	Biopsychosocial model (25)
	What are the unintentional impacts of COVID-19 public health measures on HIV transmission and early diagnosis?	
HIV treatment, linkage, and retention	How does COVID-19 affect the follow up and collection of ART drugs, prevention of mother-to-child transmission (PMTCT) services, monthly ART meetings and ART training services	Bickman's program theory or logic model (35)
	Could community-based ART groups, “pick and run strategy” and appointment spacing model be alternative options in the era of COVID-19? And are they cost-effective?	
	Are there combination therapies to COVID-19 and HIV patients	Biological component of Biopsychosocial model (25)
	Do antivirals have protective effect on COVID-19, or does HIV weaken immunity of COVID-19 patients?	
	What lessons could we learn from the four decades of unsuccessful identification of an HIV cure?	
	How can we address the cost-effectiveness and equitable distribution should COVID-19 treatment or vaccine be successful?	Social determinants of health framework (36, 37)
HIV treatment outcomes, and impacts	What is the impact of COVID-19 on the negative outcomes of HIV care and treatment or the UNAIDS-95-95-95?	UNAIDS 95-95-95 treatment targets (51)
	What does the COVID-19/HIV patient outcomes looks like in terms of social, economic, residence, and gender?	Social determinants of health framework (36, 37)
	What is the impact of stigma of variety origin on outcomes of COVID/HIV co-infected patients?	Goffman's stigma framework (55)
	What does the quality-adjusted life years (QALYs) and disability-adjusted life years (DALYs) of COVID/HIV co-infected patients look like?	WHO quality of life framework (59)
HIV care promotion and prevention services	How can we leverage the lessons on sustained behavioral change at intrapersonal level to achieve improved outcomes of COVID/HIV co-infected patients?	Health belief model (60)
	What is the role of medical mistrust and conspiracy beliefs in the success of public health interventions?	Trust and risk by Luhmann (61), Giddens (62), and Ward (63–65)
	How can we apply the integrated role of peer educators or community health workers for the prevention and promotion of HIV and COVID-19?	Structuration model of collaboration (66), Gidden's theory of structuration (67)
	What will be the role of digital health in the integrated management of HIV care continuum and spectrum of care of COVID-19?	
	How can we leverage learning's from well-implemented COVID-19 mitigation responses to the HIV pandemic?	

*ART: antiretroviral therapy; COVID-19: corona virus disease 2019; HIV: human immunodeficiency virus; WHO: World Health Organization; UNAIDS: The Joint United Nations Program on HIV/AIDS*.

## Conclusions

This commentary identifies a number of research questions for multidisciplinary specialists. The research agenda includes questions around transmission, diagnosis, treatment, prevention, and control of the HIV/COVID-19 syndemic. We have suggested several theoretical frameworks and models to guide examination of complex issues comprehensively. The outcomes of such research will hopefully provide evidence on how to effectively manage people living with HIV, diagnosed with COVID-19, or co-infected with COVID/HIV in terms of improving the prevention, promotion, and diagnosis alongside better linkage to, compliance with, and outcomes from treatment and care. We urge researchers and research funding agencies to collaborate with each other and people with lived experience to ensure these research agendas are addressed, and to further generate new questions to be identified over time. Research studies need to answer the proposed questions within a variety of contexts, including income (low-, middle-, and high-income countries), population (children, adult, older age and most-at-risk population), culture, education, and other variables. Our HIV/COVID-19 syndemic research agenda complements other recently published COVID-19 research agendas, and together we hope more integrated and complex research can eventuate. Although this commentary suggests numerous research questions, along with known theories and frameworks, we acknowledge these may not be feasible in practice—the so-called “self-fulfillment prophecy” —due to resource limitations. Despite this, we believe our proposed research agenda provides a guide for researchers and research funders to explore new and innovative areas to address both pandemics.

## Author Contributions

HG, LM, JS, KW, and PW conceived the idea. HG drafted the manuscript. All authors critically reviewed and approved the final version of the manuscript.

## Conflict of Interest

The authors declare the research was conducted in the absence of any commercial or financial relationships that could be construed as a potential conflict of interest.
